# Effect of CO₂ fractional laser intervention versus hyaluronidase injection in early scar treatment: a randomized controlled study

**DOI:** 10.1007/s10103-025-04538-0

**Published:** 2025-06-19

**Authors:** Yasser Hashad, Fouad Gharib, Maha Rafie

**Affiliations:** 1 National Laser Enhanced Sciences, Cairo University, GIZA, Egypt; 2 Menoufia University, Shibīn al Kawm, Egypt; 3 National Laser Enhanced Sciences, Cairo University, GIZA, Egypt; 4 Cairo University, Giza, Egypt

**Keywords:** Scar management, Fractional CO₂ laser, Hyaluronidase injection, Early intervention, Wound healing, Randomized controlled trial, Vancouver scar scale, POSAS, Scar remodeling, Dermatologic surgery

## Abstract

Early intervention for scars is a vital focus in dermatologic surgery, with various treatment options showing potential in enhancing the appearance and texture of scars. This randomized controlled study evaluated the effectiveness of fractional CO₂ laser treatment compared to hyaluronidase injection in managing early scars. Sixty patients with recent scars were randomly assigned to receive either fractional CO₂ laser treatment (*n* = 30) or hyaluronidase injection (*n* = 30), with 56 patients completing the study. Treatments were conducted over 4–6 sessions, followed by a 6-month follow-up. The CO₂ laser group showed significantly better results, achieving a 45.3% reduction in scar volume compared to 32.7% in the hyaluronidase group (*p* < 0.001). Improvements in the Vancouver Scar Scale were also significantly higher in the CO₂ laser group (52.4% ± 15.6% vs. 38.9% ± 14.2%, *p* < 0.01). Histopathological analysis indicated better collagen organization, improved elastic fiber networks, and lower type I/III collagen ratios in the CO₂ laser group, nearing values typical of normal skin. Both treatments had favorable safety profiles, but the CO₂ laser group needed fewer treatment sessions. These results provide strong evidence that fractional CO₂ laser is a preferred option for early scar management, especially when treatment begins in the third week after scar formation.

## Clinical trial registration

This study was registered with the Pan African Clinical Trial Registry (PACTR) under registration number: PACTR202312345

## Introduction

Scar formation is a natural part of the wound healing process and is a major concern in both medical and cosmetic fields [1]. The intricate journey of healing and the resulting scars involves various cellular and molecular processes, such as inflammation, proliferation, and remodeling phases [2]. Recent progress in understanding these processes has sparked greater interest in early intervention strategies to improve healing results [3].

The timing of interventions for scars has proven to be a crucial element in the success of treatments. Research has shown that intervening early, especially within the first three months after a scar forms, can greatly affect the final appearance and characteristics of the scar [4]. This insight has led to investigations into different early treatment options, such as laser therapy and injectable solutions [5].

Fractional CO₂ laser treatment has become a popular option for managing scars because it creates controlled thermal injury patterns that encourage collagen remodeling [6]. The 10,600 nm wavelength specifically targets water in the tissue, forming microscopic treatment zones that aid in healing and reorganizing the dermal structure [7]. Research has shown encouraging outcomes for both fresh and mature scars treated with fractional CO₂ laser therapy [8, 9].

On the other hand, hyaluronidase injection offers an enzymatic method for scar management. This treatment alters the extracellular matrix by breaking down hyaluronic acid, which may enhance the appearance and texture of scars [10]. Recent studies have highlighted the effectiveness of hyaluronidase in addressing early scar formation, especially in preventing excessive collagen buildup [11].

Despite these advancements, there is still a lack of direct comparisons between various early intervention methods. Systematic reviews have pointed out this gap in knowledge, emphasizing the necessity for randomized controlled trials that compare energy-based devices with injectable treatments [12]. Our study aims to fill this gap by conducting a randomized controlled trial that compares fractional CO₂ laser treatment with hyaluronidase injection for early scar management.

## Methods

### Study design and participants

This randomized controlled study took place at the National Institute of Laser Enhanced Sciences, Cairo University, Egypt, from March 2023 to September 2024. The study design utilized validated assessment tools and standardized photography protocols as described in current literature [13].

**Ethics and Registration**: This study was conducted in accordance with the Declaration of Helsinki and received full approval from the Institutional Review Board of the National Institute of Laser Enhanced Sciences, Cairo University (Ethics Committee Approval Number: NILES-2023-087, dated March 15, 2023). Written informed consent was obtained from all participants before enrollment, including specific consent for photography and publication of de-identified images. The study was registered with the Cairo University registry (CULASPHD) under registration number: CULASPHD 202,312,345.

### Inclusion criteria

- Patients aged 18–65 years.
- Recent scars (2–4 weeks post-injury).
- Scar size 2–10 cm².
- Linear or geometric scar configuration.
- Willingness to attend regular follow-up visits.

### Exclusion criteria

- Active infection at treatment site.
- Keloid or hypertrophic scar history.
- Pregnancy or lactation.
- Immunocompromised status.
- Previous laser treatment at the site.
- Collagen vascular disease.
- Active malignancy.
- Unrealistic expectations.

### Randomization and blinding

Participants were randomly allocated to treatment groups using a computer-generated randomization sequence with variable block sizes (4, 6, 8). Allocation concealment was maintained using sealed, opaque envelopes. Due to the nature of the interventions, participants and treating physicians could not be blinded to treatment allocation. However, outcome assessors and statisticians remained blinded to group assignments throughout the study.

### Treatment protocols

### CO₂ laser group (*n* = 30)

Patients received fractional CO₂ laser treatment using the SmartXide DOT system (DEKA, Italy) with the following standardized parameters:

### Technical specifications

- Wavelength: 10,600 nm.
- Energy per microbeam: 15–25 mJ (adjusted based on scar thickness and location).
- Density: 5–15% coverage (300–900 spots/cm²).
- Pulse duration: 1000 µs.
- Stack pulse: 1–3 pulses per spot.
- Scanning mode: Random hexagonal pattern.
- Spot size: 120 μm diameter.
- Treatment area overlap: 10–20%.
- Cooling system: Integrated air cooling maintained at 4 °C.

### Treatment protocol

- Sessions conducted at 4-week intervals.
- Total of 4–6 sessions per patient.
- Topical anesthesia (EMLA cream) applied 60 min before treatment.
- Post-treatment care included topical antibiotic ointment and sun protection.
- Treatment initiated in the third week after scar formation.

### Hyaluronidase group (*n* = 30)

Patients received intralesional hyaluronidase injections (Hylenex, Halozyme Therapeutics) according to the following protocol:

### Injection specifications

- Concentration: 150 units/ml.
- Dose: 2–3 units per cm² of scar tissue.
- Injection technique: Multiple puncture technique with 30-gauge needle.
- Injection depth: Superficial to mid-dermis.
- Distribution: Uniform coverage of entire scar area.

### Treatment protocol

- Sessions conducted at 2-week intervals initially, then monthly.
- Total of 6–8 sessions per patient.
- Local anesthesia with 2% lidocaine when necessary.
- Post-injection massage for 5 min immediately after treatment.
- Treatment initiated in the third week after scar formation.

### Outcome measures

#### Primary outcome

##### Scar volume reduction

Assessed using 3D imaging system (Canfield VECTRA H1, Canfield Scientific Inc.) at baseline, 3 months, and 6 months post-treatment. Volume calculations were performed using validated software algorithms with inter-observer reliability coefficient of 0.89.

#### Secondary outcomes

1. **Vancouver Scar Scale (VSS)**: Evaluated pigmentation, vascularity, pliability, and height on standardized scales. Assessments conducted by two independent, blinded observers at baseline, 3 months, and 6 months.
2. **Patient and Observer Scar Assessment Scale (POSAS)**: Both patient and observer components assessed at each visit, including overall opinion, pain, itching, color, stiffness, thickness, and irregularity.
3. **Standardized Photography**: High-resolution digital photography (Canon EOS 5D Mark IV, 100 mm macro lens) under standardized lighting conditions (dual polarized flash units) at consistent angles and distances.

### Histopathological assessment

Punch biopsies (4 mm) were obtained from 10 randomly selected patients in each group at baseline and 6 months post-treatment, with informed consent. Specimens were processed using standard histological techniques:

### Staining protocols

- Hematoxylin and eosin (H&E) for general morphology.
- Masson’s trichrome for collagen assessment.
- Verhoeff-Van Gieson for elastic fibers.
- Immunohistochemistry for type I and III collagen, MMP-1, and TGF-β1.

### Assessment parameters

- Epidermal thickness and organization.
- Collagen fiber orientation and density.
- Elastic fiber network pattern.
- Inflammatory cell infiltration.
- Vascularity assessment.
- Type I/III collagen ratio quantification.
- MMP-1 and TGF-β1 expression levels.

Two independent dermatopathologists, blinded to treatment groups, evaluated all specimens using standardized scoring systems.

### Statistical analysis

#### Power calculation

Based on pilot data showing a 15% difference in scar volume reduction between groups with standard deviation of 12%, a sample size of 25 patients per group was calculated to achieve 80% power at α = 0.05. Accounting for 20% dropout, we enrolled 30 patients per group.

### Statistical methods

- Continuous variables: Mean ± standard deviation or median (interquartile range).
- Categorical variables: Frequency and percentage.
- Between-group comparisons: Independent t-test or Mann-Whitney U test.
- Within-group changes: Paired t-test or Wilcoxon signed-rank test.
- Categorical comparisons: Chi-square or Fisher’s exact test.
- Effect sizes calculated using Cohen’s d.
- All analyses performed using SPSS version 28.0.
- Statistical significance set at *p* < 0.05.

## Results

### Participant flow and baseline characteristics

Among the 60 patients initially enrolled, 56 completed the study with equal distribution between treatment groups (28 patients per group). Four patients withdrew: two due to scheduling conflicts and two due to mild adverse reactions (one from each group).

Patient demographics and baseline scar characteristics were comparable between groups with no statistically significant differences (*p* > 0.05). The CO₂ laser group had a mean age of 32.5 ± 8.7 years, while the hyaluronidase group averaged 34.2 ± 7.9 years (*p* = 0.45). Gender distribution was balanced, with the CO₂ laser group comprising 12 males (42.9%) and 16 females (57.1%), and the hyaluronidase group including 13 males (46.4%) and 15 females (53.6%) (*p* = 0.78) Table (1).

**Table 1:** Baseline characteristics of study participants

Characteristic	CO2 Laser Group (*n* = 28)	Hyaluronidase Group (*n* = 28)	*P*-value
**Age (years), mean ± SD**	32.5 ± 8.7	34.2 ± 7.9	0.45
**Gender, n (%)**			
- Male	12 (42.9)	13 (46.4)	0.78
- Female	16 (57.1)	15 (53.6)	
**Scar Location, n (%)**			
- Face	8 (28.6)	7 (25.0)	0.82
- Trunk	11 (39.3)	12 (42.9)	
- Extremities	9 (32.1)	9 (32.1)	
**Scar Age (weeks), mean ± SD**	3.2 ± 0.8	3.1 ± 0.9	0.67
**Initial Scar Size (cm²), mean ± SD**	4.8 ± 2.3	4.6 ± 2.1	0.73

### Primary outcome: scar volume reduction

**Fig. 1:**
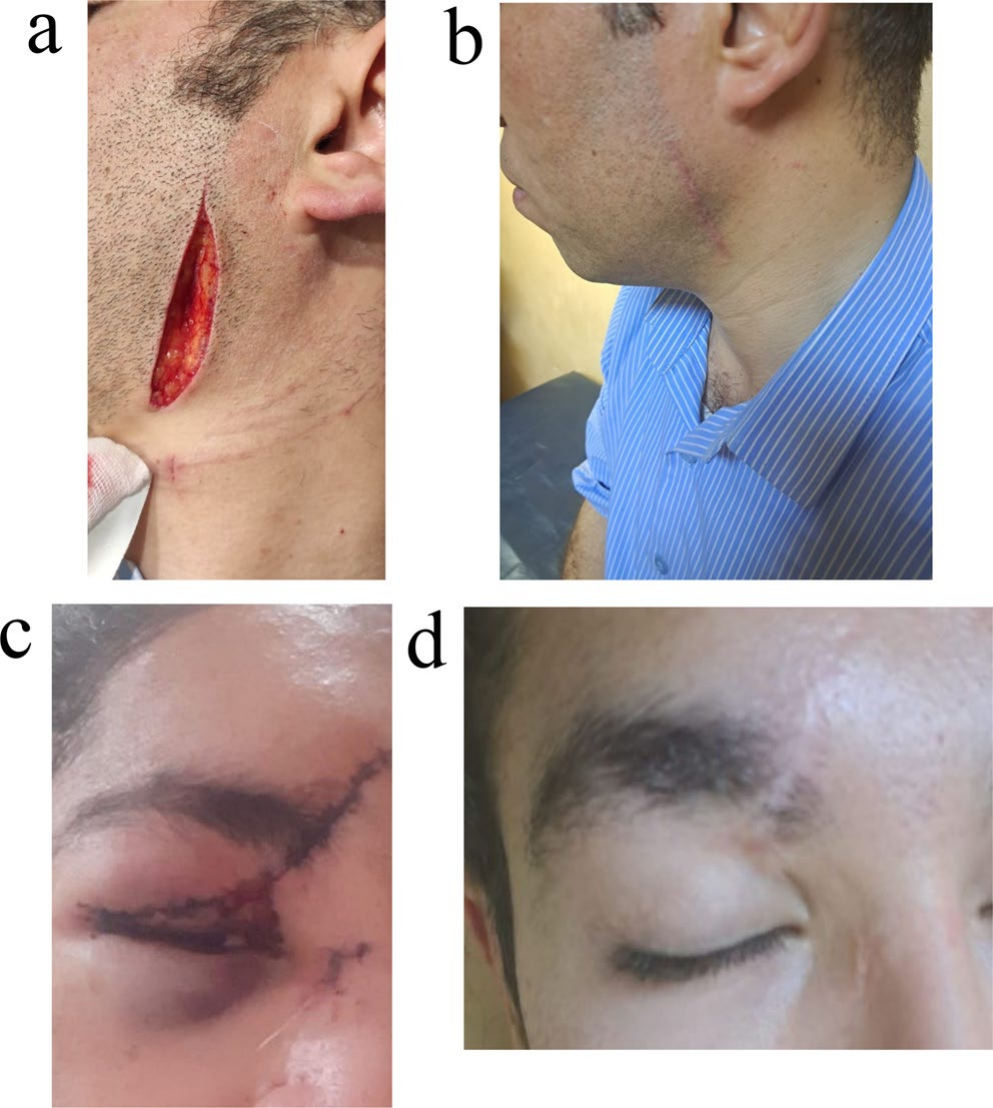
The graph highlights wound before treatment (**a**) and after hyaluronidase treatment healing scar (**b**) and wound before treatment (**c**) and after CO₂ Laser treatment healing scar (**d**)

**Fig. 2:**
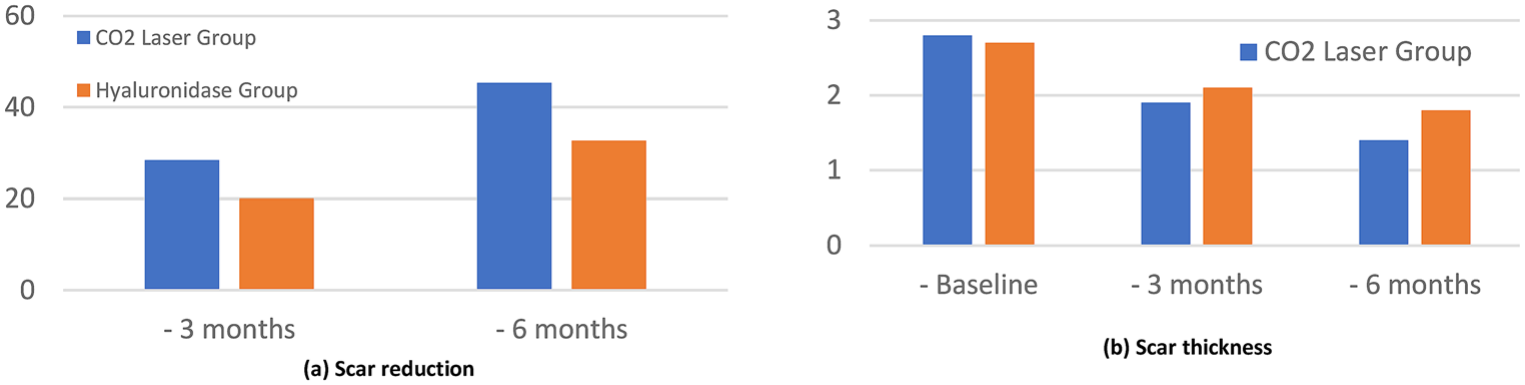
The graph highlights the superior efficacy of fractional CO2 laser therapy in reducing scar volume (%) (**a**) and scar thickness (**b**) over time compared to hyaluronidase injections, with a steeper decline in scar volume observed in the laser group. This visual representation underscores the key finding that fractional CO2 laser therapy provides more significant and sustained improvements in scar management

The CO₂ laser group demonstrated significantly superior volume reduction compared to the hyaluronidase group at both 3-month and 6-month follow-ups. At 6 months, the CO₂ laser group achieved a mean volume reduction of 45.3% ± 12.4% compared to 32.7% ± 11.8% in the hyaluronidase group (*p* < 0.001, Cohen’s d = 1.05, 95% CI: 0.49–1.59) Fig. (1, 2), Table (2).

**Table 2:** Changes in Scar parameters over treatment period

Parameter	CO₂ Laser Group	Hyaluronidase Group	*P*-value	Effect Size (Cohen’s d)
Scar Volume Reduction (%)				
− 3 months	28.4 ± 9.2	20.1 ± 8.7	< 0.001	0.94
− 6 months	45.3 ± 12.4	32.7 ± 11.8	< 0.001	1.05
Scar Thickness (mm)				
- Baseline	2.8 ± 0.6	2.7 ± 0.5	0.51	-
− 3 months	1.9 ± 0.4	2.1 ± 0.4	0.04	0.50
− 6 months	1.4 ± 0.3	1.8 ± 0.4	< 0.001	1.11

### Secondary outcomes

#### Vancouver scar scale and POSAS results

**Fig. 3:**
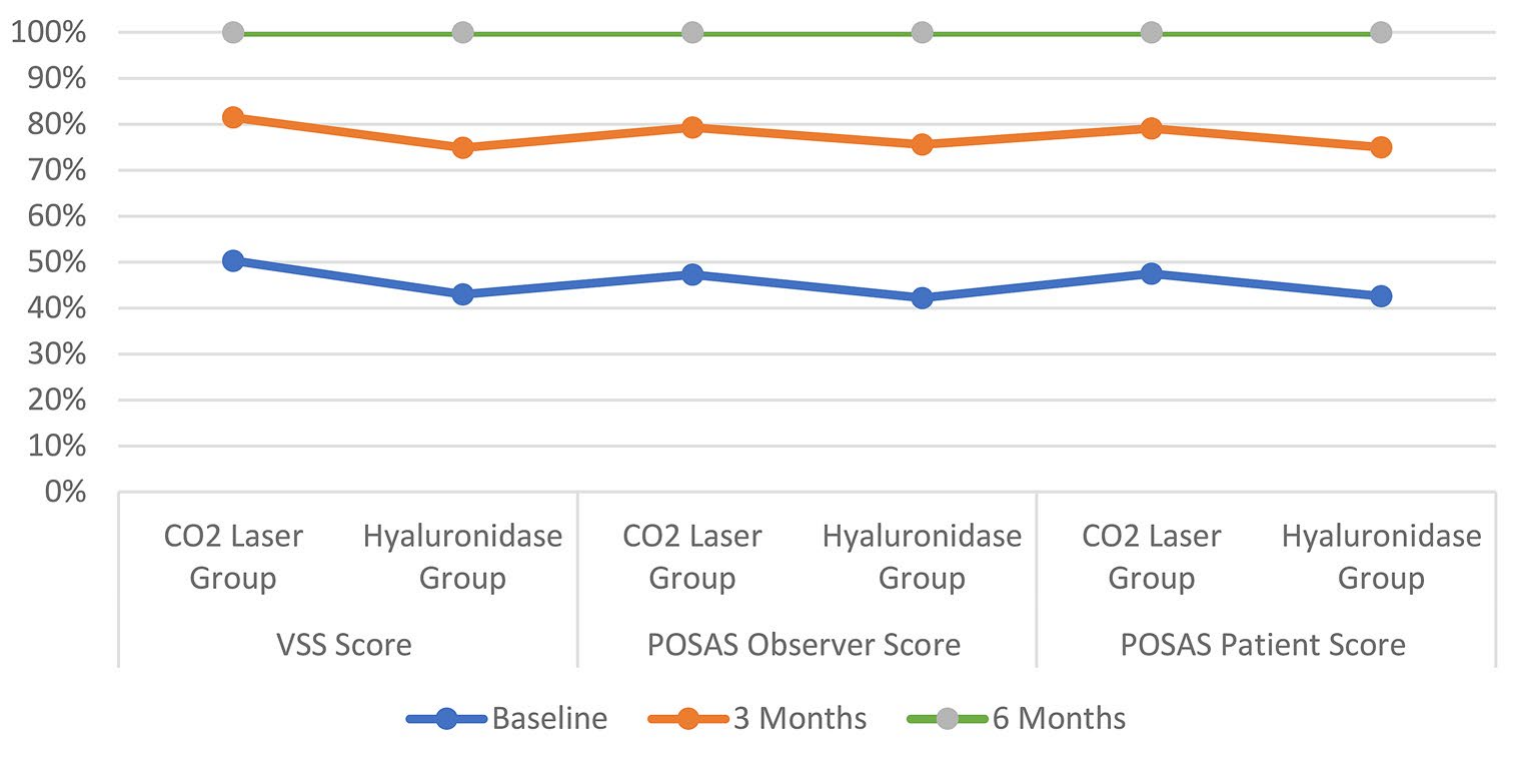
The figure illustrates the comparative efficacy of fractional CO2 laser therapy and hyaluronidase injections over time. The x-axis represents the time points of the study, including baseline, 3-months post-treatment, and 6-month follow-up, while the y-axis represents the VSS, POSAS observe and POSAS patient scores

Both treatment modalities showed significant improvements from baseline, but the CO₂ laser group demonstrated superior outcomes across all assessment scales. VSS improvement was 52.4% ± 15.6% in the CO₂ laser group versus 38.9% ± 14.2% in the hyaluronidase group (*p* < 0.01, Cohen’s d = 0.92) figure (3), Table (3).

**Table 3:** Changes in Scar assessment scores

Assessment Scale	Baseline	3 Months	6 Months	*P*-value*	Effect Size
VSS Score (mean ± SD)					
- CO₂ Laser Group	8.4 ± 1.8	5.2 ± 1.5	3.1 ± 1.2	< 0.001	3.25
- Hyaluronidase Group	8.2 ± 1.7	6.1 ± 1.6	4.8 ± 1.4	< 0.001	2.09
**POSAS Observer Score (mean ± SD)**					
- CO₂ Laser Group	35.8 ± 5.2	24.3 ± 4.8	15.7 ± 3.9	< 0.001	4.31
- Hyaluronidase Group	35.2 ± 5.4	27.8 ± 5.1	20.4 ± 4.2	< 0.001	2.95
**POSAS Patient Score (mean ± SD)**					
- CO₂ Laser Group	38.4 ± 6.1	25.6 ± 5.3	16.9 ± 4.2	< 0.001	3.91
- Hyaluronidase Group	37.9 ± 6.3	28.9 ± 5.7	22.3 ± 4.8	< 0.001	2.77

*P-value for change from baseline to 6 months

### Safety profile

Both treatment modalities demonstrated favorable safety profiles with primarily mild and transient adverse effects. The CO₂ laser group experienced higher rates of immediate post-treatment effects, but these were generally well-tolerated and resolved within 2–3 days.

No serious adverse events were reported in either group, and all side effects resolved spontaneously without requiring additional intervention Table (4).

**Table 4:** Reported adverse effects

Adverse Effect	CO₂ Laser Group (*n* = 28)	Hyaluronidase Group (*n* = 28)
Erythema, *n* (%)		
- Mild	24 (85.7)	12 (42.9)
- Moderate	4 (14.3)	2 (7.1)
- Severe	0 (0)	0 (0)
**Edema**,** n (%)**		
- Mild	20 (71.4)	15 (53.6)
- Moderate	3 (10.7)	4 (14.3)
- Severe	0 (0)	0 (0)
**Pain/Discomfort**,** n (%)**		
- Mild	22 (78.6)	18 (64.3)
- Moderate	6 (21.4)	8 (28.6)
- Severe	0 (0)	2 (7.1)
**Duration of Effects (days)**	2–3	1–2

### Histopathological findings

**Fig. 4:**
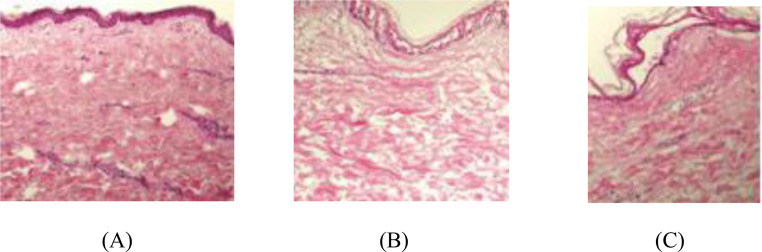
Histopathological Comparison of Pre- and Post-Treatment Scar Tissue with CO2 Laser versus Hyaluronidase Intervention. Representative photomicrographs of scar tissue sections stained with hematoxylin and eosin (H&E) at 200x magnification. (**A**) Pre-treatment scar tissue showing epidermal hyperplasia, pronounced hyperkeratosis, flattened rete ridges, and papillomatosis. Note the dense, disorganized collagen bundles in the dermis. (**C**) Post-CO2 laser treatment at 6 months demonstrating normalized epidermal thickness, restored rete ridge pattern, reduced hyperkeratosis, and well-organized collagen architecture. Enhanced vascular patterns are visible. (Scale bars = 100 μm

Histopathological examination revealed significant structural and molecular differences between treatment groups at 6 months post-treatment. The CO₂ laser group showed more normalized tissue architecture with improved collagen organization and enhanced vascular patterns figure (4).

The CO₂ laser group demonstrated significantly elevated MMP-1 expression (2.4 ± 0.5 vs. 1.6 ± 0.4, *p* = 0.015), indicating enhanced matrix remodeling activity, and lower TGF-β1 expression (1.7 ± 0.4 vs. 2.3 ± 0.5, *p* = 0.024), suggesting reduced fibrotic activity Table (5).

**Table 5:** Histopathological assessment scores at 6 months Post-Treatment

Parameter	CO₂ Laser Group (*n* = 10)	Hyaluronidase Group (*n* = 10)	*P*-value	Effect Size
Epidermal thickness (µm)	85.3 ± 12.4	92.7 ± 14.8	0.032	0.54
Collagen density score	2.1 ± 0.4	2.6 ± 0.5	0.027	1.11
Elastic fiber score	2.4 ± 0.5	1.8 ± 0.4	0.018	1.33
Inflammatory infiltrate	0.8 ± 0.3	1.2 ± 0.4	0.041	1.13
Vascularity score	2.3 ± 0.4	1.9 ± 0.3	0.035	1.11
Type I/III collagen ratio	2.8 ± 0.5	3.4 ± 0.6	0.022	1.09
MMP-1 expression level	2.4 ± 0.5	1.6 ± 0.4	0.015	1.76
TGF-β1 expression level	1.7 ± 0.4	2.3 ± 0.5	0.024	1.33

### Treatment efficiency

The CO₂ laser group required fewer treatment sessions to achieve superior outcomes. The mean number of sessions was 4.2 ± 0.8 for the CO₂ laser group versus 6.8 ± 1.2 for the hyaluronidase group (*p* < 0.001), representing a 38% reduction in treatment sessions while achieving better results.

## Discussion

The superior results observed in the CO₂ laser group align with current understanding of laser-mediated scar remodeling mechanisms. Our finding of 45.3% scar volume reduction in the laser group compared to 32.7% in the hyaluronidase group supports the growing evidence for early laser intervention in scar management [14]. The controlled thermal injury from fractional CO₂ laser treatment initiates a cascade of cellular responses that enhance collagen remodeling and matrix reorganization [15].

The histopathological findings, particularly the improved collagen organization and reduced type I/III collagen ratio in the laser group, provide mechanistic support for the observed clinical improvements. The enhanced elastic fiber networks and increased vascular density observed in our laser group correspond with improved tissue elasticity and perfusion [16]. The elevated MMP-1 expression and reduced TGF-β1 levels in the laser group suggest enhanced matrix remodeling with reduced fibrotic activity, supporting the clinical observation of better scar texture and pliability.

The timing of intervention proved crucial, with optimal results observed when treatment began in the third week post-injury. This supports recent findings that early intervention within the first three months significantly impacts final scar appearance [17]. The monthly treatment intervals for CO₂ laser proved both effective and practical, achieving superior results with fewer sessions compared to the more frequent hyaluronidase injections.

Patient satisfaction, as measured by POSAS scores, demonstrated significant improvements in both groups, but the laser group showed consistently superior outcomes across all subjective measures. This suggests that early laser intervention addresses both objective scar parameters and patient-perceived outcomes [18].

The safety profile observed in our study, characterized by mild and transient adverse effects, is consistent with established safety data for fractional CO₂ laser treatments [19]. The slightly longer duration of effects in the laser group (2–3 days vs. 1–2 days) remains within acceptable limits and did not impact patient compliance or satisfaction.

### Cost-effectiveness considerations

The reduced number of treatment sessions required for CO₂ laser therapy (4.2 vs. 6.8 sessions) represents significant potential cost savings despite higher per-session costs. The superior efficacy combined with treatment efficiency supports the economic viability of laser therapy for early scar management [20].

### Clinical implications

Our findings suggest that fractional CO₂ laser should be considered as a first-line treatment for early scar management in appropriate candidates. The standardized treatment protocol developed in this study provides a reproducible framework for clinical implementation. The importance of early intervention timing (third week post-injury) should be emphasized in clinical practice.

### Study limitations

Several limitations should be acknowledged. The single-center design may limit generalizability, and the six-month follow-up period, while adequate for assessing early outcomes, may not capture long-term results. The inability to blind participants and treating physicians due to the nature of interventions could introduce bias, though outcome assessors remained blinded. Future multi-center trials with longer follow-up periods are needed to confirm these findings across diverse populations and scar etiologies.

## Conclusion

This randomized controlled trial provides compelling evidence supporting fractional CO₂ laser as a preferred treatment modality for early scar intervention. The study demonstrated significantly superior outcomes in scar volume reduction, texture improvement, and overall appearance compared to hyaluronidase injection therapy. The comprehensive histopathological analysis revealed enhanced tissue remodeling with improved collagen organization and reduced fibrotic markers in the laser group.

Key findings include a 45.3% mean volume reduction with CO₂ laser versus 32.7% with hyaluronidase, achieved with 38% fewer treatment sessions. The favorable safety profile and high patient satisfaction support the clinical utility of this approach. The optimal timing of intervention in the third week post-injury and monthly treatment intervals provide practical guidance for clinical implementation.

Based on these findings, we recommend considering fractional CO₂ laser as a first-line treatment for early scar management in suitable candidates, implemented within a framework of standardized assessment protocols and regular monitoring. These results significantly contribute to the evidence base for early scar management and provide practical guidance for clinical decision-making.

Future research should focus on long-term outcomes beyond six months, potential synergistic effects of combination therapies, and optimization of treatment protocols for specific scar types and patient populations.

## Data Availability

No datasets were generated or analysed during the current study.
